# Retrospective Glances

**Published:** 1876-12

**Authors:** 


*Retrospective Glances.*—To turn the dioramic pages of the past, produces the psychological anomaly of opposite emotions at the same time in the human heart. Besides the intangible and indefinable charm, that shimmers like a pearly mist about the thought, or word—"memory;" perhaps there is no period of the by-gone we can recall, but is fraught with the antipodal feelings of pain and pleasure. One picture of the shifting scenes is full of sunshine, and makes the heart, at its recall, thrill with purest joy—and now, perhaps, there follows, after a painted memory, around which hangs a pall-like darkness: the shadow of a great sorrow, the veil of sadness, or the haunting spectre of vain regret.

The life of no rational being can be so barren that he finds nothing in the past he would fain make immortal or would have blotted out forever. Thus it is that though time may rob the eye of its early lustre, the step of its youthful buoyancy, and weave silver threads among the golden or raven woof of our hair, he is a constant reviver of the soul's memorials until the mnemonical faculty is lost in the imbecility of extreme old age. He stands beside us amid the incidental gloom of our present powers, and, like a psychological necromancer, reproduces in immortal youth the freshness and charm of the past, as he touches the heart with the magical wand of memory, and makes it quiver under the spell of some joyful episode of our lives now buried, but not hidden, in the crypt of the past.

Nearly six years ago we ventured THE MEDICAL RECORD upon the favor and generosity of the profession. These are but few annual revolutions of this old, old world, and yet how fraught they have been with important movements in the ever progressive spirit of the age. Each record of these years shows to us grand achievements in letters, art, science, and every department of mental or mechanical science. The great "art of healing" has not lagged behind in the onward march. Its important revelations, during these consecutive years, have more fully demonstrated the fact that the practice of medicine is a simple, yet profound and comprehensive science, and has shown in glaring colors its shameful abuses by charlatans who have crept into the arena of the medical fraternity, until the people and the profession are now, more than ever, deeply impressed withthe important truth, that the "ministry of medicine" should no longer be left to empiricism, but to a thorough investigation and intelligent knowledge of the science of medicine, its skillful adaptation to the great and perfect law of nature, and a sympathetic, earnest ministration to suffering humanity. When this, "a consummation devoutly to be wished," shall take place, without one ignoble exception, then may we hope to see the profession moving as a shining orb in its truly legitimate sphere, universally respected and honored, and proving a blessing, but in never an instance a bane to mankind.

Since the first publication of THE RECORD, we are happy to say, it has been most kindly received and generously supported by the profession, and, with few exceptions, our position as senior editor and business manager has been very pleasant during these past years. Some few subscribers, not more than eight or ten, have written to us rather sharply when we pressed them for the subscription price of THE RECORD, and a few others, when we failed to promptly answer all their communications.

We can but think, though, if they had considered that it is more difficult for us to settle a bill of two hundred dollars per month than for them to pay the small sum of three dollars subscription money for such a journal as THE RECORD, or that among scores of letters to write, or answer, it was impossible for us to reply promptly to all at once, they would have been more lenient towards our importunities for money, and to remissness in our correspondence. All who know us well will say that those who have treated us unkindly do not know us, or that they are hopeless cases. We believe it is because they do not know us, and feel satisfied that they will renew their subscriptions for 1877, and give us their support and sympathy.

Dr. R. C. Word has consented to take charge of our business department for the year 1877, which will give us more time to do our part in the editorial department, and the general get up of the journal. Under our new arrangement no one will have any cause to complain, as Dr. Word has recently taken an office at a convenient point to ours, and promises to give every subscriber the attention they desire without delay. Our friend and co-editor, Dr. W. T. Goldsmith, will continue his valuable assistance. To our associate editors and co-laborers at a distance, who have aided us, we are very grateful, and trust that they will not fail to continue their valuable contributions to our pages, and to use their influence and efforts to enlarge our circulation.

In conclusion, we again proffer our heartfelt thanks for the courtesy, kindness and cordial encouragement we have received in the past years from our patrons and the profession at large. With the hope that the *entente cordiale* may continue in the future, and with many kind wishes we turn our glance from the past annual cycle of time, and greet our subscribers and friends with a very happy new year. P.

